# Temporal Trends in the Prevalence of Dementia in South Korea

**DOI:** 10.1093/geroni/igab046.990

**Published:** 2021-12-17

**Authors:** Jung Hyun Kim, Anja Leist

**Affiliations:** 1 University of Luxembourg, University of Luxembourg, Diekirch, Luxembourg; 2 University of Luxembourg, Esch-sur-Alzette, Diekirch, Luxembourg

## Abstract

Background. Secular decreases in the prevalence of cognitive impairment and dementia have been observed in several Western countries, however, few systematic investigations of temporal trends in dementia have been conducted in South Korea. Method. Data came from N=8,006 individuals (N=2,110 assessed twice) aged 65 years and older participating in the Korean Longitudinal Study of Aging 2008 and 2018. Dementia was indicated by a score ≤ 17 on the Korean Mini-Mental State Examination (K-MMSE). Dementia was regressed on the year of survey, adjusting for multiple demographic and socio-economic confounders, and, in additional models, also chronic diseases and lifestyle factors related to health, social, and religious activities. Results. Across waves, the share of individuals with low socio-economic status decreased. The prevalence of chronic diseases, including diabetes, heart diseases, stroke, and psychiatric diseases, increased over time. Alcohol consumption increased, whereas smoking rates, religious affiliation, and participation in religious activities decreased. Controlling for all covariates and compared to 2008, we observe decreases in dementia prevalence in 2018 by 52% (2018: OR 0.48, CI 0.42, 0.56). Women’s MMSE scores were more than two times as likely as men’s to indicate dementia (OR 2.59, CI 2.15, 3.14). Discussion. Decreases in dementia prevalence in Korea are partly attributable to improved socio-economic conditions and can be observed despite the increased prevalence of chronic conditions. However, secular trends were not fully explained by these and lifestyle factors. We discuss further individual-level and contextual-level mechanisms that may have contributed to these findings.

